# Multitemporal SAR Image Despeckling Based on a Scattering Covariance Matrix of Image Patch

**DOI:** 10.3390/s19143057

**Published:** 2019-07-11

**Authors:** Xiaoshuang Ma, Penghai Wu

**Affiliations:** 1 Department of Resources and Environmental Engineering, Anhui University, Hefei 230601, China; 2 Anhui Province Key Laboratory of Wetland Ecosystem Protection and Restoration, Anhui University, Hefei 230601, China

**Keywords:** synthetic aperture radar, speckle filtering, multitemporal, nonlocal means, covariance matrix

## Abstract

This paper presents a despeckling method for multitemporal images acquired by synthetic aperture radar (SAR) sensors. The proposed method uses a scattering covariance matrix of each image patch as the basic processing unit, which can exploit both the amplitude information of each pixel and the phase difference between any two pixels in a patch. The proposed filtering framework consists of four main steps: (1) a prefiltering result of each image is obtained by a nonlocal weighted average using only the information of the corresponding time phase; (2) an adaptively temporal linear filter is employed to further suppress the speckle; (3) the final output of each patch is obtained by a guided filter using both the original speckled data and the filtering result of step 3; and (4) an aggregation step is used to tackle the multiple estimations problem for each pixel. The despeckling experiments conducted on both simulated and real multitemporal SAR datasets reveal the pleasing performance of the proposed method in both suppressing speckle and retaining details, when compared with both advanced single-temporal and multitemporal SAR despeckling techniques.

## 1. Introduction

The synthetic aperture radar (SAR) remote sensing technique has attracted wide attention since the launch of the advanced spaceborne sensors. These SAR satellites or sensors can provide images of the observed areas in different time phases in all day time and all weather conditions, and work in different imaging modes. The above advantages make the SAR a good tool to observe the Earth. However, speckle noise is inherent in SAR systems, as with most of the active imaging systems, which degrades the quality of the SAR data and hinders most applications using SAR data. In the imaging step of certain SAR systems, a multilook procedure is deployed to suppress the amount of speckle noise, which can, however, result in severe spatial resolution loss. Therefore, the application of a speckle reduction technique is often essential before using SAR data, especially for most satellite radar systems which provide single-look data. This paper focuses on the despeckling of single-polarization SAR data; fully polarimetric SAR or interferometric SAR despeckling is outside the scope of this paper.

During the past four decades, a number of single-temporal SAR despeckling methods were proposed. In the early years, many SAR filters were developed based on minimum mean-square error theory [1], such as the Lee filter [2] and the Kuan filter [3]. Local filters that work in the wavelet domain [4,5] and homomorphic approaches [6,7] that take the log of the data before applying the denoising process were also widely studied. However, most of these traditional despeckling techniques can hardly obtain a good balance between preserving the fine image structure and effectively reducing speckle, although they generally have high processing efficiency.

In the last ten years, the nonlocal means (NLM) scheme [8] has attracted the interest of researchers when developing SAR speckle reduction methods [9,10,11]. The main idea of the NLM filter is to search similar image patches and to use a weighted average to estimate the true value of the reference pixel based on the similarity between the reference and the sample. Recently, inspired by NLM, Parrilli et al. [12] proposed a SAR block-matching denoising method and 3D filtering method (SAR-BM3D). This method achieves a remarkable despeckling performance by employing a collaborative filtering strategy conducted in the wavelet domain, using the similarity and redundancy information of nonlocal patches. It needs to be pointed out that the deep learning theory has been a hot topic in remote sensing applications in recent years [13,14,15], and has also been applied to filter SAR data [16].

Thanks to the newly launched satellite radar systems, multitemporal SAR images of the same area are now available in many cases. Theoretically, when dealing with the despeckling issue of multitemporal images, exploiting both the temporal and spatial information can provide better results than the single-temporal filters. In recent years, some multitemporal SAR filters have been investigated. In 2014, Su et al. [17] proposed a two-step multitemporal NLM filter, which conducts a nonlocal filtering step driven by the redundancy information in the temporal domain, and then employs nonlocal estimation in the spatial domain. Chierchia et al. [18] extended the SAR-BM3D method to filter multitemporal images by exploiting more temporal information to guide both the similar patch search and image filtering steps. More recently, Zhao et al. [19] proposed a unified framework which can extend some well-known single-image despeckling techniques to filter multitemporal images, based on the ratio image between the noisy image and the temporally averaged image. For further details of the multitemporal despeckling methods presented in the early years, we refer the reader to a review paper written by Trouvé et al. [20].

For most single-polarization SAR filters, to estimate the true values of the intensity or amplitude data, only the statistical information of the intensity or amplitude is considered. However, for single-look complex (SLC) SAR data, the phase information is available and can also help to characterize the scattering traits of the targets. Consider the phase information when despeckling the amplitude data can provide better processing results. Inspired by this idea, this paper presents a multitemporal SAR filter for SLC data, which is developed under the NLM framework. One of the main innovations of this work is that the filtering framework is based on a scattering covariance matrix (SCM) constructed by the complex data of the pixels in each image patch. By doing so, both the statistical information of the amplitude of each pixel and the phase difference between any two pixels in a patch are utilized to guide the filtering process. In addition, differing from most multitemporal SAR filters that use a change detection approach to exploit the temporal redundancy, the proposed method utilizes an adaptive temporal filter based on the stationarity of the patches, which can avoid the issue of determining the change detection threshold.

In the first step of the proposed method, the basic estimations of the patches of each image in the different time phases are independently estimated by a nonlocal weighted average. Then, by searching and grouping the similar patches in time and obtaining the stationarity information of the patch groups, a simple but effective temporal linear filter is used to further suppress the speckle level. In the final step, another weighted average is employed to obtain the final estimation of the patches, and an aggregation process is used to tackle the multiple estimations issue for each pixel.

The rest of this paper is organized as follows. The idea of the scattering covariance matrix of patch is introduced in Section 2. In Section 3, the multitemporal SAR despeckling framework is presented. Then, the good filtering performances of the proposed method are validated by the experiments conducted on both simulated and real multitemporal SAR datasets in Section 4, followed by conclusions in Section 5.

## 2. The Scattering Covariance Matrix of Each Image Patch

### 2.1. The Covariance Matrix of SAR Data

It needs to be noted that, although this paper focuses on the despeckling issue of single-polarization SAR data, it is still necessary to recall the statistical trait of fully polarimetric SAR (PolSAR) data, since the idea of SCM employed in the proposed method stems from it.

For single-look PolSAR data, the polarimetric information of a pixel can be characterized by a polarimetric covariance matrix (PCM), which is constructed by the outer product of the scattering vector α of this pixel with its conjugate transpose $\alpha^{K}$ [21]:

(1) $$\begin{array}{cc} C & =\alpha \alpha^{K}\\ & ={\left[S_{\mathrm{HH}},\;\sqrt{2}S_{\mathrm{HV}},\;S_{\mathrm{VV}}\right]}^{T}{\left({\left[S_{\mathrm{HH}},\;\sqrt{2}S_{\mathrm{HV}},\;S_{\mathrm{VV}}\right]}^{T}\right)}^{K}\\ & =\left[\begin{array}{ccc} |S_{\mathrm{HH}}{|}^{2} & \sqrt{2}S_{\mathrm{HH}}S_{\mathrm{HV}}^{*} & S_{\mathrm{HH}}S_{\mathrm{VV}}^{*}\\ \sqrt{2}S_{\mathrm{HV}}S_{\mathrm{HH}}^{*} & 2|S_{\mathrm{HV}}{|}^{2} & \sqrt{2}S_{\mathrm{HV}}S_{\mathrm{VV}}^{*}\\ S_{\mathrm{VV}}S_{\mathrm{HH}}^{*} & \sqrt{2}S_{\mathrm{VV}}S_{\mathrm{HV}}^{*} & |S_{\mathrm{VV}}{|}^{2} \end{array}\right] \end{array}$$

with

(2) $$S_{\mathrm{HV}}=|S_{\mathrm{HV}}|\exp(j\phi_{\mathrm{HV}})$$

where $\left|S_{\mathrm{HV}}\right|$ and $\phi_{\mathrm{HV}}$ denote, respectively, the amplitude and the phase of the vertical transmitting (subscript “V”) and horizontal receiving (subscript “H”) polarization channel; j is the imaginary part; and the superscripts T and * are, respectively, the transpose operator and the conjugate operator. Clearly, the off-diagonal terms in the PCM can reflect the phase differences between the different polarizations, and thus, the PCM can better characterize the scattering traits of the targets, compared with the polarimetric scattering vector α.

To compare the structure similarity between two image patches, only intensity or amplitude information is considered in most NLM-based SAR filters. A direct problem is that, as mentioned in the introduction, the phase information in SLC SAR data can also help to characterize the scattering traits of the targets and considering such information when comparing patches can provide better results. As revealed in [22], considering the homogeneity similarity between patches as well as the structure similarity is useful in improving the performances of the NLM-based filters.

Based on the above analyses and inspired by the PCM of PolSAR data, we propose to use the SCM to represent the complex scattering information of all the pixels in an image patch. Assuming that all the pixels in a patch centered at pixel *x* are statistically independent, we introduce a scattering vector for this patch by jointing the back-scattering signals of all the pixels:

(3) $$v_{x}=\left[S_{1},S_{2},\cdots ,S_{n}\right]$$

where $S_{n}=|S_{n}|\exp(j\phi_{n})$ denotes the complex data of the *n*th pixel. In this paper, we fix the size of all the patches as 3×3 (i.e., n=9). The reason is that, the computational time of the filtering procedure could increase more than 10 times if we enlarge the patch size from 3 × 3 to 5 × 5, and we found small patches are helpful to retain point targets which is meaningful for many target detection applications. It can be seen that, this joint vector has a similar form with the PolSAR scattering vector α, with the difference that it is constructed by the scattering information of different pixels rather than by the scattering information of different polarimetric channels of a certain pixel. Therefore, intuitively, we can construct the SCM for this patch as the way of constructing PCM for PolSAR data, which is:

(4) $$\begin{array}{cc} M_{x} & =v_{x}v_{x}^{K}\\ & =\left[\begin{array}{cccc} A_{11} & A_{12} & \cdots & A_{1n}\\ A_{21} & A_{22} & \cdots & A_{2n}\\ \cdots & \cdots & \cdots & \cdots \\ A_{n1} & A_{n2} & \cdots & A_{nn} \end{array}\right] \end{array}$$

where each element is $A_{ab}=S_{a}S_{b}^{*}=|S_{a}||S_{b}|\;\exp(j(\phi_{a}-\phi_{b}))$. Clearly, each diagonal term of M is the intensity of a pixel, while each off-diagonal term contains the phase difference between two pixels in the same patch, as well as the amplitude information of each pixel. By doing so, the issue of comparing the similarity of two patches can be converted into comparing the similarity between two SCMs, so that more information can be exploited rather than only using the amplitude information.

It is well known that the PCM of PolSAR data follows a complex Wishart distribution [23]. As we can see, since each element of the joint scattering vector v follows a complex Gaussian distribution as in the PolSAR scattering vector α, the SCM of a patch can also be modeled by the complex Wishart distribution given the clean matrix z, as follows:

(5) $$P(M|z)=\frac{L^{qL}|M{|}^{L-q}\exp\{-L\mathrm{Tr}(z^{-1}M)\}}{Q(L,q)|z|}$$

with

(6) $$Q(L,q)=\pi^{q(q-1)/2}\prod_{i=1}^{q}\Gamma (L-i+1)$$

where Tr(⋅) denotes the trace of the matrix, Γ(⋅) denotes the Gamma function, *L* is the number of looks, and q=n is the dimension of the SCM M.

### 2.2. Similarity Measures Based on the SCM

Under the NLM framework, the proposed method is developed using the SCM of an image patch as the basic processing unit. Hence, the first question is how to measure the similarity between two patches based on their SCMs. To handle this issue, we employ a likelihood-ratio test method designed for complex Wishart-distributed data [24]. If we assume that two SCMs, $M_{x}$ and $M_{y}$, follow the same complex Wishart distribution (i.e., *x* and *y* have the same true SCM), then the test statistic for the single-look data can be derived as:

(7) $$F(M_{x},M_{y})=2^{2n}\frac{\left|M_{x}|\cdot |M_{y}\right|}{|M_{x}+M_{y}{|}^{2}}$$

Taking the logarithm of Equation (7), we have:

(8) $$G_{1}(M_{x},M_{y})=2n\ln2+\ln|M_{x}|+\ln|M_{y}|-2\ln|M_{x}+M_{y}|$$

where $G_{1}(M_{x},M_{y})$ is non-positive and is equal to zero when two patches have the same true values.

The idea of the likelihood-ratio test for two patches can be extended to measure the stationarity of a patch group (i.e., the equality of multiple variables). With the hypothesis that all the patches of a group are equal, the test statistic for *t* variables can be derived as [25,26]:

(9) $$F(M_{x}(1),M_{x}(2),\cdots ,M_{x}(t))=\frac{\prod_{i=1}^{t}\left|M_{x}(i)\right|}{|R{|}^{t}}$$

with the temporal average

(10) $$R=\sum_{i=1}^{t}M_{x}(i)/t$$

The value of this multivariable test statistic is between zero and one, and the more similar the patches are, the closer the value is to 1.

## 3. The Proposed Multitemporal SAR Despeckling Framework

**Figure 1:**
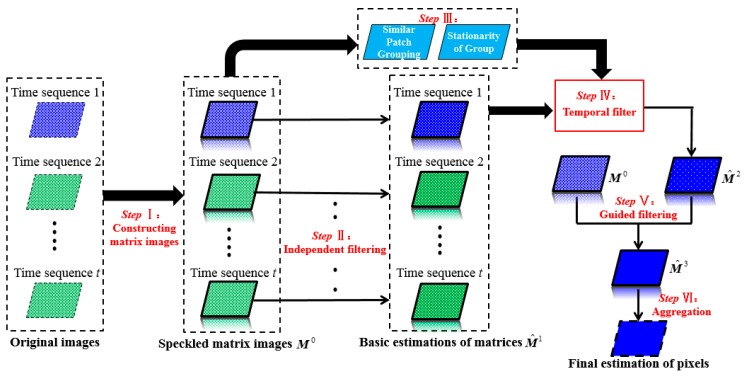
The schematic map of the proposed multitemporal synthetic aperture radar (SAR) despeckling method. As an example, the image of time sequence 1 is considered as the filtering target in this map.

The schematic map of the proposed SCM-based multitemporal SAR (SCM-MSAR) filter is shown in Figure 1. In Step I, we construct the SCMs for all patches of all time sequences (see Equations (3) and (4)), by doing so, new images with each unit represented as a matrix is obtained, which is termed as “matrix image” in the rest of this paper. Then, in Step II, each matrix image is independently filtered to obtain its basic estimation. In Step III, to utilize redundancy information in time, similar patches in different times are grouped and the stationarity information of each group is obtained (see Equations (9) and (10)). After this, a temporal linear filter is employed to further reduce the speckle level of the target matrix image in Step IV. In Step V, the final estimation of the target matrix image is obtained by a guided filter, using both the information of the speckled data and the temporally filtered data. Lastly, since each pixel is included in several patches, and an aggregation step is taken to tackle the multiple estimations problem. More details of the proposed SCM-MSAR filter are provided in the following.

### 3.1. The First Spatial Filtering Approach

The SCM-MSAR filter includes two spatial filtering steps (Step II and Step V in Figure 1), which are weighted average approaches conducted on the speckled and the temporally filtered matrix images, respectively. In the first spatial filtering step, we use the following linear formulation to filter patch x in each time sequence independently:

(11) $${\hat{M}}_{x}^{1}=\sum_{i=1}^{w_{1}\times w_{1}}W_{i}M_{i}^{0}/\sum_{i=1}^{w_{1}\times w_{1}}W_{i}$$

with the weight:

(12) $$W_{i}=\exp\left(-{\left(G_{1}(M_{x}^{0},M_{i}^{0})/h_{1}\right)}^{2}\right)$$

where $w_{1}\times w_{1}$ denotes the size of the large filtering window centered at *x*; $h_{1}$ is the weight normalization parameter. As shown in Figure 1, the different superscripts of M represent the estimation results of the matrix image in the different filtering steps, which is consistent in the whole paper. $G_{1}(\cdot )$ is the similarity function between two speckled SCMs calculated by Equation (8). In fact, we can observe that, this is a standard NLM filter, with the exception that the intensities of all the pixels in a patch (i.e., the diagonal terms of the SCM) are filtered in each weighted average step.

### 3.2. Temporally Similar Patch Grouping

In some cases, due to the limited precision of the georeference or the geocoding process, the multitemporal SAR images will not be exactly registered, which leads to a slight offset in the space between SAR images. In such cases, if we take a temporal filter simply using the pixels located in the same image position of different times, the image could be badly blurred. In this paper, to facilitate the temporal filtering procedure, we propose a method to search and group similar patches in different times. It should be pointed out that the similar patch grouping approach is employed to utilize redundancy information in time, rather than performing accurate registration.

For a given patch *x* in the target time, and in its local window with the size of w×w, we can compare the similarity between *x* and each patch in the window of any complementary time by Equation (8). By doing so, the most similar pixel with respect to *x* in all the time sequences is obtained. However, we found that the patch grouping process is not good enough due to the influence of speckle, and considering the information of the neighbors of *x* can refine the estimation process (i.e., we conduct the above grouping process for all the patches inside a 3 × 3 local window of *x*), and find the patch in each time which is considered as the similar patch most frequently.

**Figure 2:**
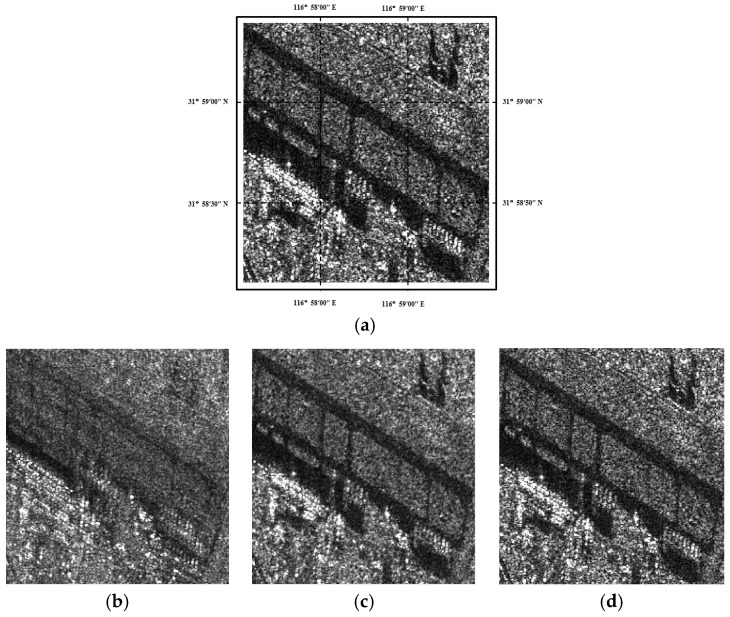

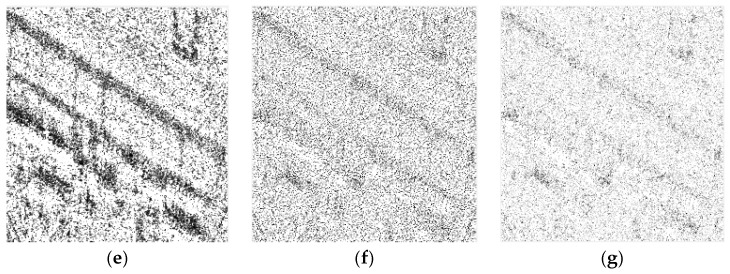
Temporally averaged results for a multitemporal SAR dataset with three images: (**a**) one of the speckled images. (**b**–**d**) Directly averaged result, and the averaged results with the similar patch grouping method presented in [17] and the proposed method, respectively. (**e**–**g**) The ratio images for (**b**–**d**), respectively.

Figure 2 shows the temporal average of a multitemporal SAR dataset with and without similar patch grouping, and we also compared the temporal average results by using the proposed similar patch grouping approach and the one presented in [17]. To reveal the feasibility of different approaches, we display the ratio images between the original speckled image and the temporal averaged images. Due to the multiplicative trait of speckle, the content residing in the ratio image should be very like pure speckle, if the temporal averaged approach reduces the speckle level without blurring the details. Clearly, the original result is badly blurred, while the results with the similar patch grouping approaches, especially with the proposed one, better retain the details.

### 3.3. Temporal Filtering Approach

Taking into account the redundant information in the temporal domain when designing, multitemporal SAR filters can achieve better results than single-temporal filters. To achieve this goal, most of the current multitemporal SAR filters use a change detection step, often followed by a simple temporal averaging using the unchanged pixels, to further suppress the speckle or to compare the similarity between pixels more precisely. As we stated before, the phase information is not considered in these methods. Furthermore, determination of a proper threshold for change detection is difficult in many cases.

In this paper, we propose a simple but effective temporal filter based on the stationarity of a similar patch group in time (Step IV in Figure 1), which is depicted as:

(13) $${\hat{M}}_{x}^{2}=J_{x}\cdot R+(1-J_{x})\cdot {\hat{M}}_{x}^{1}$$

where $J_{x}$ denotes the stationarity calculated by Equation (9), and R is the temporal average of the basic estimations of the patches. As we can see, when $J_{x}\approx 1$ (i.e., high stationarity), ${\hat{M}}_{x}^{2}\approx R$; and when $J_{x}\approx 0$ (i.e., high nonstationary), ${\hat{M}}_{x}^{2}\approx {\hat{M}}_{x}^{1}$. This implies that the proposed temporal filtering step is adaptive, which is more applicable and reasonable than using a change detection step based on a threshold, and simply taking a temporal average.

### 3.4. The Second Spatial Filtering Approach

In the second spatial filtering step (Step V in Figure 1), we employ a similar framework used in the SAR guided filters [27,28] to reduce the noise residing in the temporally filtered image of the target time sequence. The main idea is to guide the filtering process with the help of the original data. In other words, we consider the information of both the temporally filtered patches and the speckled patches in the target time, which is formulated as:

(14) $${\hat{M}}_{x}^{3}=\sum_{i=1}^{w_{1}\times w_{1}}W_{i}{\hat{M}}_{i}^{2}/\sum_{i=1}^{w_{1}\times w_{1}}W_{i}$$

with the weight

(15) $$W_{i}=\exp\left(-{\left(G_{1}(M_{x}^{0},M_{i}^{0})\cdot G_{2}({\hat{M}}_{x}^{2},{\hat{M}}_{i}^{2})/h_{2}\right)}^{2}\right)$$

where $h_{2}$ is another weight normalization parameter, and $G_{2}({\hat{M}}_{x}^{2},{\hat{M}}_{i}^{2})$ denotes the similarity between two patches in the temporally filtered image. It needs to be noted that, since the assumption of a complex Wishart distribution does not still hold in the filtered image, this similarity measure term cannot be calculated by Equation (8). Considering that the SCMs are Hermitian positive definite matrices, we use the affine-invariant metric proposed by D’Hondt et al. [29] to measure their similarity:

(16) $$G_{2}({\hat{M}}_{x}^{2},{\hat{M}}_{i}^{2})=\mathrm{Tr}[{\left({\hat{M}}_{x}^{2}\right)}^{-1}{\hat{M}}_{i}^{2}+{\hat{M}}_{x}^{2}{\left({\hat{M}}_{i}^{2}\right)}^{-1}-2n]$$

For the setting of the weight normalization parameters $h_{1}$ and $h_{2}$ in the spatial filtering approaches, we deploy a strategy based on the complexity of the image scene [30,31]: before each spatial filtering approach, the parameter is chosen as the 0.8-quantile of the distribution of the similarity between any two neighboring pixels computed throughout the image. By doing so, the parameters can be set lower to better preserve the image details, but when the image scene is complex, the parameter can be set larger, to better suppress the speckle.

After the second spatial filtering approach, the final estimations of all the pixels in each patch are obtained. Since each pixel is included in n patches when constructing the matrix images, we finally average the multiple estimations to obtain more robust results.

## 4. Experimental Part

In this section, to illustrate the effectiveness of the proposed SCM-MSAR filter, we describe the experiments carried out on simulated images and two real multitemporal SAR datasets, and make comparisons between the proposed filter and three state-of-the-art filters: the single-temporal SAR-BM3D filter [12], the multitemporal SAR-BM3D (MSAR-BM3D) filter [18], and the ratio-based multitemporal SAR (RB-MSAR) filter [19], which were all accomplished by the source codes offered by the authors of the respective papers. In the experiments, the sizes of the searching windows of all the methods were fixed as 35×35. For reproducibility, the SLC SAR datasets used in the experiments and the despeckling results of the SCM-MSAR filter can be downloaded from the link (http://sendimage.whu.edu.cn/en/resources/).

Generally speaking, an outstanding SAR filter should have the following characteristics [32,33]:

(1) speckle is reduced to a large degree in homogeneous areas;
(2) image features and radiometric information are well preserved; and
(3) the result should be without artifacts.

To quantify the performances of the filters in the above aspects, we use the following four indicators:

(1) Equivalent number of looks (ENL): the ENL indicator is widely used in the literature to evaluate the level of speckle, and the higher the ENL value, the better the filter reduces the speckle.
(2) Figure of merit (FOM): the FOM index [34] is used to assess the edge-preservation capability of despeckling methods. It was designed for experiments with simulated images, since a reference is needed to calculate this index. A higher FOM value indicates a better edge-preservation result.
(3) Edge-preservation degree based on the ratio of average (EPD-ROA): for the real SAR datasets, EPD-ROA [35] is one of the indices used to assess the edge-preservation capability of filters which does not need a reference. This index should be close to one if the filter shows a good edge-preservation performance.
(4) Mean of ratio (MOR): due to the statistical trait of speckle, a MOR value between the speckled and the despeckled data should be very close to one if the filter can effectively preserve the radiometric information.

### 4.1. Experiments with Simulated Datasets

In this part, we use a framework proposed by Di Martino et al. [32] to simulate the multitemporal SAR datasets. This framework was developed based on a SAR raw signal simulator (SARAS) [36], which simulates the SAR images relevant to canonical scenes. In SARAS, to evaluate a SAR signal, sound geometrical and electromagnetic models for the evaluation of the reflectivity function of the scene and a model for the transfer function of the system are employed. By doing so, proper physical models for the sensed surface, the scattering, and the radar operational mode are considered. Through simulating multiple SAR images as different instances relevant to the same scene, SAR images with an arbitrary number of looks can be obtained. Hence, to simulate a “clean” reference image, one can generate enough images of the same scene by this framework and average them.

**Figure 3:**
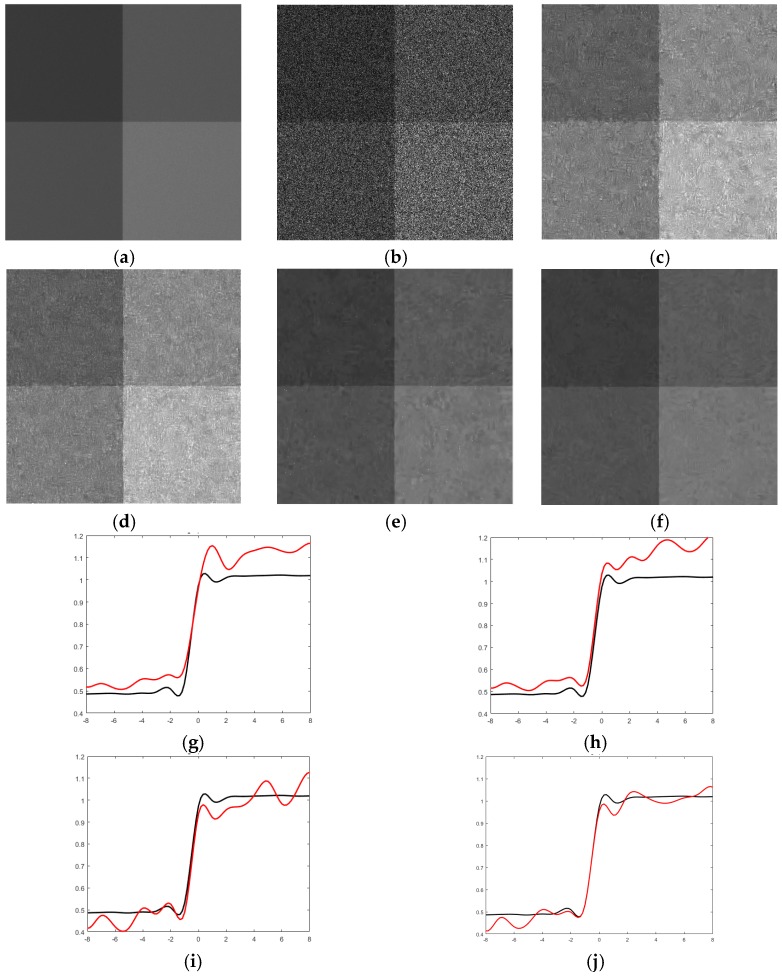
Despeckling results on the simulated multitemporal dataset having four images with r=0.4: (**a**,**b**) The noise-free and the speckled images of the target time, respectively. (**c**–**f**) The images despeckled by the single-temporal SAR-BM3D filter, the multitemporal synthetic aperture radar (MSAR)-BM3D filter, the ratio-based (RB)-MSAR filter, and the scattering covariance matrix (SCM)-MSAR filter, respectively. (**g**–**j**) The edge profiles of the images filtered by the single-temporal SAR-BM3D filter, the MSAR-BM3D filter, the RB-MSAR filter, and the SCM-MSAR filter, respectively.

First, we simulated three datasets which all have four time sequences. To inspect the influence of the changes between the images in the target time and the other times (i.e., the complementary times) on the despeckling results, we randomly chose some pixels with a certain ratio and changed their values by a mean filter before exerting speckle for each complementary time. The change ratios were set as 0.4, 0.2, and 0.1 for the three simulated datasets, respectively. The simulated images and the filtered results of the dataset with r=0.4 are shown in Figure 3. At first sight, both the single-temporal and multitemporal SAR-BM3D filters show some artifacts which blurs the image to some degree. Visually, the multitemporal SAR-BM3D filter performs slightly better than the single-temporal filter both in suppressing speckle and retaining edges. Compared with the BM3D filters, the RB-MSAR filter smooths the homogeneous areas to a greater degree, but some strong speckle still resides on the image. The superiority of the SCM-MSAR filter is distinguished: the speckle is reduced by a large degree and the boundaries are effectively preserved, and moreover, with much less artifacts. To visualize the capability of the despeckling methods in preserving edges, we plotted the edge profiles for each filtered image (Figure 3). The conclusions we can draw from the edge profiles are generally in line with the above observation.

The above analyses are backed up by the quantitative assessment results listed in Table 1. The SCM-MSAR filtered image has the highest FOM and ENL values among all the despeckled images. Furthermore, the MOR values reveal that the proposed method shows the best performance in retaining radiometric information, while the single-temporal SAR-BM3D filter distorts the radiometric information to a much larger degree. An interesting phenomenon, which should be pointed out, is that as the change ratio decreases, the superiority of the SCM-MSAR filter with regard to the other two multitemporal filters becomes less significant. This reveals that, for a certain dataset, the superiority of the proposed method will be more significant if the image scene is highly dynamic in time, benefiting from the adaptive temporal filtering approach.

We also simulated a dataset having eight time sequences with r=0.1. Clearly, as the time sequences increase, the performances of all the multitemporal filters have been notably improved since more redundant temporal information is exploited. Thanks to the adaptive temporal filtering approach, the superiority of the proposed filter compared with other filters becomes more significant as the time sequences increase.

### 4.2. Experiments with Two Real Multitemporal Datasets

**Figure 4:**
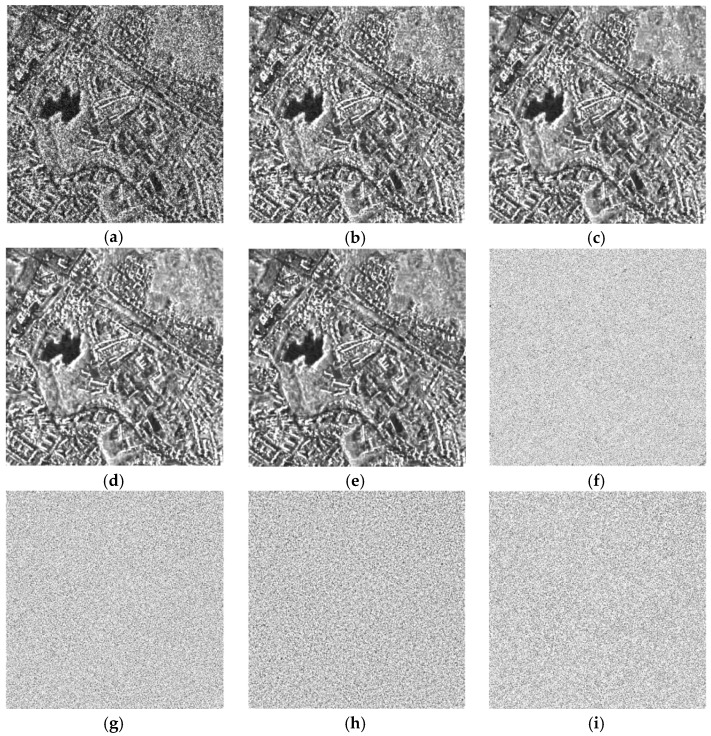
Despeckling results on the TerraSAR-X dataset with two time sequences: (**a**) the speckled image of the target time. (**b**–**e**) The images despeckled by the single-temporal SAR-BM3D filter, the MSAR-BM3D filter, the RB-MSAR filter, and the SCM-MSAR filter, respectively. (**f**–**i**) The ratio images of the single-temporal SAR-BM3D filter, the MSAR-BM3D filter, the RB-MSAR filter, and the SCM-MSAR filter, respectively.

Experiments were also carried out on two real multitemporal datasets, to verify the effectiveness of the proposed method. The first dataset has two time sequences which were acquired by the TerraSAR-X system in Ruhr, Germany, on 20 February 2008 and 4 March 2008, respectively. The imaging mode is strip mode with a spatial resolution of 3 m × 3 m working on X band, and the polarization type is HH. From Figure 4, visually, the two SAR-BM3D methods and the proposed method show quite comparable results in preserving image details, especially preserving the high returns from the urban area. Compared with the other three methods, the RB-MSAR filter shows a slight oversmoothing problem. The above observations are supported by the ratio images and the EPD-ROA values shown in Table 2. The single-temporal SAR-BM3D still does not effectively suppress the speckle, although it has the highest EPD-ROA value. The proposed method again shows the best result in preserving radiometric information.

**Figure 5:**
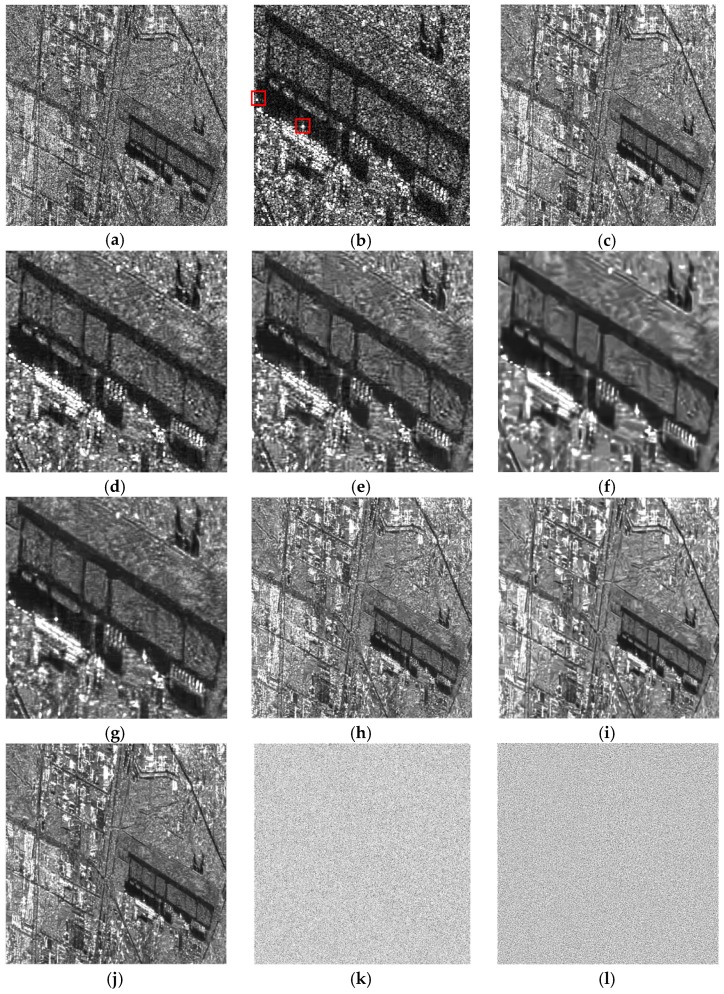

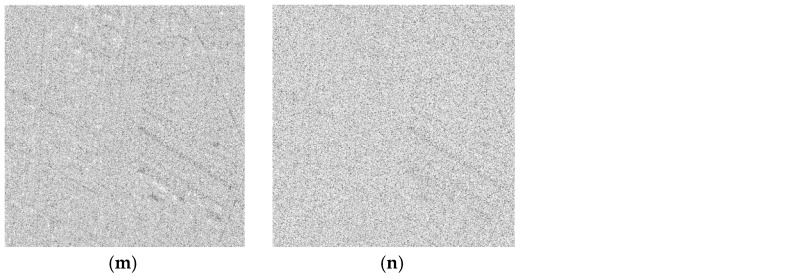
Despeckling results on the Sentinel-1 dataset with three time sequences: (**a**) the speckled image of the target time. (**b**) The sub-images cropped from (**a**). (**c**–**f**) The images despeckled by the single-temporal SAR-BM3D filter, the MSAR-BM3D filter, the RB-MSAR filter, and the SCM-MSAR filter, respectively. (**g**–**j**) The sub-images cropped from (**c**–**f**), respectively. (**k**–**n**) The ratio images of the single-temporal SAR-BM3D filter, the MSAR-BM3D filter, the RB-MSAR filter, and the SCM-MSAR filter, respectively.

The second real SAR dataset shown in Figure 5 was acquired by the Sentinel-1 system and has three images which were acquired on 31 July 2017, 17 September 2017 and 11 October 2017, respectively. The imaging mode is interferometric wide swath mode with a spatial resolution of 5 m × 20 m working on C band, and the polarization type is HV. Since the original images are not well-registered, the offset estimation step presented in Section 3 was deployed before applying each of the multitemporal filters. Once again, we can observe that RB-MSAR has an oversmoothing problem, and some artifacts are exhibited in the despeckled image, which degrades the resolution of the image to some degree. From the ENL and EPD-ROA values, the proposed method obtains a good balance between reducing speckle and retaining image features, and the MOR values again indicate the outstanding performance of the proposed method in preserving radiometric information.

**Figure 6:**
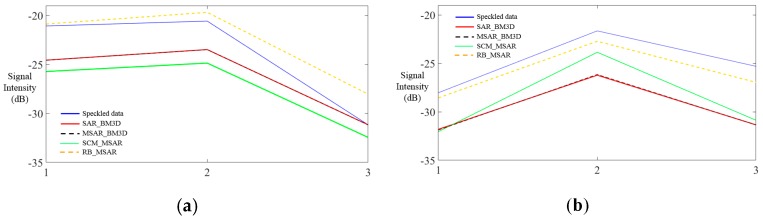
(**a**) shows the profiles of a line across a strong scatterer processed by the different methods, and (**b**) shows the profiles for the other strong scatterer.

Preservation of signals from strong scatterers is essential for target and man-made structure detection. To show the capability of the filters in retaining the information of strong scatterers, two lines were taken across two high-return targets, respectively (marked by the red rectangle in Figure 5b), and the signal intensity of the lines after despeckling by the different methods are plotted in Figure 6. Here, it can be observed that the data filtered by the proposed method are the most in line with the original data, which indicates the proposed method’s ability to retain strong targets. The other methods smear the signatures to a greater or lesser degree.

**Figure 7:**
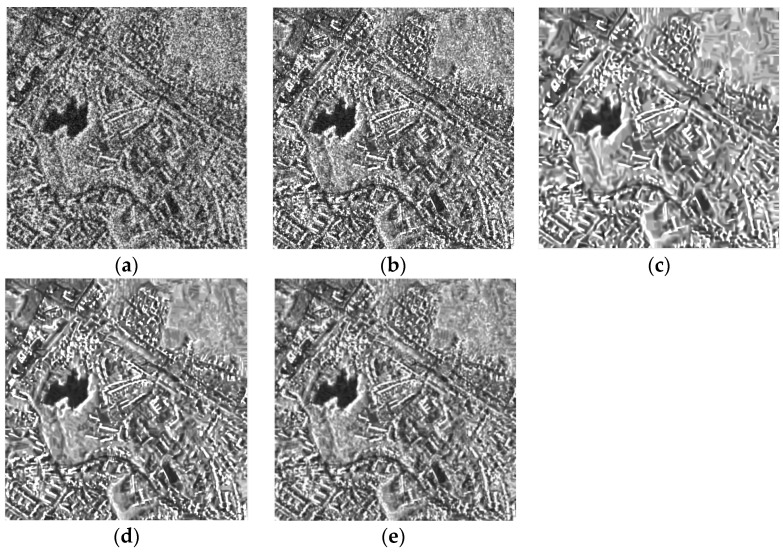
Despeckled images of the different steps of SCM-MSAR filter on the TerraSAR-X dataset: (**a**) The speckled image of the target time. (**b**–**e**) The despeckled images despeckled by the first spatial filtering step, the temporal filtering step, the second spatial filtering step, and the aggregation step, respectively.

The proposed SCM-MSAR filter consists of three filtering steps and a multiple estimations aggregation step. To reveal the influence of the different steps on the filtering result, we display the filtered image of each step in Figure 7. As we can see, after the first spatial filtering step, the speckle is suppressed to some degree. Then, by using the adaptive temporal filter, the speckle is notably reduced, but some details are slightly blurred. In the second spatial filtering step, since the information of the original speckled image is used to guide the despeckling process, the oversmoothing problem is alleviated and finally, via the multiple estimations aggregation step, the resolution of the image is further improved.

## 5. Conclusions

In this paper, a multitemporal SAR filter was proposed for the despeckling of SLC SAR data, using a SCM of each patch as the basic processing unit, which can better take advantage of the phase difference between pixels. The proposed method includes two spatial filtering approaches. One is a nonlocal filtering scheme, and the other is a guided filtering scheme. In addition, an adaptive temporal filter is employed in the proposed method based on the temporal stationarity of patches, to better exploit the redundant temporal information. We compared the proposed method with some state-of-the-art single-temporal and multitemporal SAR filters, in experiments conducted on both simulated and real datasets. The experimental results revealed that the proposed method can not only obtain a good balance between reducing speckle and retaining image structure, but also show an outstanding performance in preserving radiometric information.

It must be pointed out that, since the output of each spatial and temporal filtering step of the proposed method is the matrix image, the phase of each pixel in a patch cannot be directly retrieved using the filtered SCM; that is to say, the proposed filter only tackles with the despeckling issue of amplitude or intensity. Our future work will focus on the despeckling problem of both amplitude and phase for complex SAR data.

## Figures and Tables

**Table 1:** Quantitative assessment results for the simulated datasets filtered by the different methods.

		ENL	FOM	MOR
	**SAR-BM3D**	51.6	0.807	0.822
r=0.4 t=4	**MSAR-BM3D**	55.2	0.836	0.833
	**RB-MSAR**	66.6	0.855	0.904
	**SCM-MSAR**	**68.1**	**0.881**	**0.912**
r=0.2 t=4	**MSAR-BM3D**	58.8	0.846	0.866
	**RB-MSAR**	69.5	0.883	0.918
	**SCM-MSAR**	**70.2**	**0.909**	**0.925**
r=0.1 t=4	**MSAR-BM3D**	60.4	0.861	0.881
	**RB-MSAR**	**73.0**	0.910	0.930
	**SCM-MSAR**	72.1	**0.917**	**0.932**
r=0.1 t=8	**MSAR-BM3D**	100.1	0.909	0.935
	**RB-MSAR**	116.0	0.921	0.960
	**SCM-MSAR**	**122.3**	**0.939**	**0.987**

ENL: equivalent number of looks; FOM: figure of merit; MOR: mean of ratio. The quantitative assessment numbers in bold denote the best results among the three different filtering methods

**Table 2:** Quantitative assessment results for the real multitemporal Datasets Filtered by the Different Methods.

	TerraSAR-X Dataset			Sentinel-1 Dataset		
	ENL	EPD-ROA	MOR	ENL	EPD-ROA	MOR
**SAR-BM3D**	18.3	**0.69**	0.885	18.1	**0.67**	0.877
**MSAR-BM3D**	30.1	0.67	0.856	27.9	0.65	0.859
**RB-MSAR**	**45.3**	0.62	0.852	**33.0**	0.59	0.853
**SCM-MSAR**	44.6	0.65	**0.904**	31.5	0.64	**0.911**

EPD-ROA: edge-preservation degree based on the ratio of average.
